# A Qualitative Transcriptional Signature for the Risk Assessment of Precancerous Colorectal Lesions

**DOI:** 10.3389/fgene.2020.573787

**Published:** 2021-01-15

**Authors:** Qingzhou Guan, Qiuhong Zeng, Weizhong Jiang, Jiajing Xie, Jun Cheng, Haidan Yan, Jun He, Yang Xu, Guoxian Guan, Zheng Guo, Lu Ao

**Affiliations:** ^1^Collaborative Innovation Center for Chinese Medicine and Respiratory Diseases Co-Constructed by Henan Province & Education Ministry of P.R. China, Academy of Chinese Medical Sciences, Henan University of Chinese Medicine, Zhengzhou, China; ^2^Key Laboratory of Medical Bioinformatics, Key Laboratory of Ministry of Education for Gastrointestinal Cancer, School of Basic Medical Sciences, Fujian Medical University, Fuzhou, China; ^3^Department of Colorectal Surgery, The Affiliated Union Hospital of Fujian Medical University, Fuzhou, China

**Keywords:** qualitative transcriptional signature, incidence risk score, colorectal cancer, ulcerative colitis, colorectal adenoma

## Abstract

It is meaningful to assess the risk of cancer incidence among patients with precancerous colorectal lesions. Comparing the within-sample relative expression orderings (REOs) of colorectal cancer patients measured by multiple platforms with that of normal colorectal tissues, a qualitative transcriptional signature consisting of 1,840 gene pairs was identified in the training data. Within an evaluation dataset of 16 active and 18 inactive (remissive) ulcerative colitis subjects, the median incidence risk score of colorectal carcinoma was 0.6402 in active ulcerative colitis subjects, significantly higher than that in remissive subjects (0.3114). Evaluation of two other independent datasets yielded similar results. Moreover, we found that the score significantly positively correlated with the degree of dysplasia in the case of colorectal adenomas. In the merged dataset, the median incidence risk score was 0.9027 among high-grade adenoma samples, significantly higher than that among low-grade adenomas (0.8565). In summary, the developed incidence risk score could well predict the incidence risk of precancerous colorectal lesions and has value in clinical application.

## Introduction

Colorectal cancer (CRC) is one of the commonest malignancies worldwide, with high morbidity and mortality rates (Arnold et al., 2017). The condition mainly develops from malignant transformation of acquired precancerous lesions (Conteduca et al., 2013; Brenner et al., 2014), such as inflammatory bowel disease (IBD) and colorectal adenomas. Chronic IBD is a major type of precancerous colorectal lesions, which has two forms: ulcerative colitis (UC) and Crohn’s disease (CD). Long-term exposure to chronic inflammation is the primary risk factor for CRC pathogenesis (Axelrad et al., 2016; Fujita et al., 2018). The progression of CRC ranges from the physiological state to quiescent chronic inflammation that then progresses to active chronic inflammation without dysplasia. Dysplasia eventually develops and progresses to outright malignancy (Bjerrum et al., 2014). Moreover, the proportion of bowel affected by IBD and the severity of inflammation likewise affect CRC risk of patients with IBD (Feagins et al., 2009). Another major type of precancerous colorectal lesions is colorectal adenomas (Conteduca et al., 2013; Brenner et al., 2014). The development sequence is from normal colonic mucosa to small tubular adenoma to large adenoma and finally to cancer (Levine and Ahnen, 2006). The risk of developing colorectal cancer for patients with adenomas is two to four times higher than those patients without adenomas (Lotfi et al., 1986; Avidan et al., 2002; Rex et al., 2006). Individuals suffering signs and symptoms suggestive of CRC, including IBD, changes in bowel habits, and bloody stools are advised to seek medical help, including endoscopic or radiologic imaging examination and non-invasive tests (Quaife et al., 2014; Mhaidat et al., 2018). Colonoscopy is an essential step in the diagnosis of CRC with 4–21% inaccurate rate (Stanciu et al., 2007; Huang et al., 2016), which is mainly influenced by the image quality and the endoscopists’ experience (Costamagna and Marchese, 2010; Kaminski et al., 2010; Tumino et al., 2017). Currently, established non-invasive tests, such as the fecal occult blood test (FOBT), have a low sensitivity (Schottinger et al., 2016) and positive predictive value (Azimafousse Assogba et al., 2015; Brown et al., 2018). Methylated Septin9 (^m^SEPT9) has superior sensitivity compared to FOBT, which range from 36.6 to 95.6% (Warren et al., 2011; Ahlquist et al., 2012; Toth et al., 2012; Lee et al., 2013; Church et al., 2014). Moreover, some serum protein biomarkers, including carcinoembryonic antigen, CA19.9, and CA125, are used for monitoring the prognosis of CRC patients (Duffy et al., 2007; Soler et al., 2016). However, patients can be identified as either cancerous or non-cancerous based on colonoscopy or these diagnostic signatures (Guan et al., 2018). None could accurately assess the risk of cancer incidence among non-cancerous patients with precancerous colorectal lesions. And, to the best of our knowledge, there is currently no such molecule-based incidence risk score. Thus, it is of great clinical value to construct a molecular signature to assess the incidence of precancerous colorectal lesions converting to CRC, which could eventually aid in the prevention of CRC occurrence.

Our recent studies (Ao et al., 2018; Guan et al., 2018) demonstrated that compared with the quantitative transcriptional signatures, the qualitative transcriptional signatures—namely the relative expression orderings (REOs) of gene pairs within individual samples—are robust against experimental batch effects and could be directly applied to samples at the individualized level (Guan et al., 2016; Ao et al., 2018). In addition, we have reported that the qualitative transcriptional signatures are also highly robust against specimens with different proportions of tumor epithelial cells from different tumor locations from the same patients (Cheng et al., 2017), partial RNA degradation during specimen preparation and storage (Chen et al., 2017) and amplification bias for minimum specimens (Liu et al., 2017). The qualitative transcriptional signature is thus much more applicable to clinical application. Furthermore, the qualitative transcriptional signature is also suitable for application to inaccurately sampled specimens in clinical settings (Ao et al., 2018; Guan et al., 2019).

Based on the unique advantages of the qualitative transcriptional signature, we selected gene pairs with stable but reversal REO patterns among CRC and normal colorectal tissues as the signature for calculating incidence risk scores of precancerous lesions. These scores, in turn, were used to predict the incidence risk of malignant transformation among non-cancer patients suffering such precancerous colorectal lesions. Score performance was evaluated using multiple independent datasets via the comparison of CRC incidence risk scores among non-cancer patients with precancerous lesions (i.e., UC and adenomas) at different disease stages. Results revealed that the incidence risk scores of high-grade precancerous lesions were significantly higher than those of low-grade lesions, suggesting that the signature could well predict the incidence risk of CRC in patients suffering precancerous colorectal lesions.

## Materials and Methods

### Data and Preprocessing

The gene expression profiles used in this study were shown in Tables 1, 2, including CRC samples and normal samples. All normal colorectal tissue samples were obtained from individuals that were demonstrated to lack polyps and a known family history of previous CRC. Notably, all UC and adenoma tissue samples analyzed in this study were obtained from biopsy. All data were downloaded from Gene Expression Omnibus (Barrett et al., 2013) (GEO^1^) and The Cancer Genome Atlas (International Cancer Genome Consortium et al., 2010) (TCGA^2^).

**TABLE 1:** Datasets used for building the CRC incidence-risk score.

Platform	GEO Acc	Normal	GEO Acc	Cancer
Affymetrix	GSE4107	10	GSE27854	115
	GSE9348	12	GSE21510	123
	GSE47908	15	GSE17536	177
	GSE36807	7	GSE10714	7
	GSE10714	3	GSE4183	15
	GSE4183	8	GSE9348	70
			GSE32323	17
			GSE33113	90
Illumina RNA_seq	GSE48634	69	GSE37178	84
	GSE56789	40	GSE33126	9
	GSE53306	12	GSE31279	44
			GSE50760	36
			TCGA	556

*Empty spaces indicate that there is no sample in the corresponding category.*

**TABLE 2:** Datasets used for evaluating the performance of the score.

Platform	GEO Acc	Active UC	Inactive UC	UC_inflammation	UC_without_inflammation
**Affymetrix**	GSE13367	16	18		
	GSE9452			8	13
**RNA_seq**	GSE53306	16	12		
		**High-grade adenoma**	**Low-grade adenoma**		
**Affymetrix**	GSE37364	13	16		
	GSE8671	10	14		
		**Normal**	**CRC**		
**Affymetrix**	GSE22619	10			
**Affymetrix PrimeView^TM^Array**	Our_Data1		33		
**Illumina HiseqXten**	Our_Data2		13		

*Empty spaces indicate that there was no sample in the corresponding category; Active UC and Inactive UC denote samples with active and inactive (remissive) ulcerative colitis, respectively; UC_inflammation and UC_without_inflammation denote samples with and without macroscopic signs of inflammation, respectively; High-grade adenoma and Low-grade adenoma denote colorectal adenoma samples with high-grade and low-grade dysplasia, respectively. Normal denotes the normal colorectal tissue samples were obtained from individuals that were demonstrated to lack polyps and a known family history of previous CRC; Our_Data1 and Our_Data2 respectively denotes 33 CRC biopsy and 13 CRC surgical resection specimens measured in our previous study.*

For array-based data measured using the Affymetrix platform, raw mRNA expression data (.CEL files) were downloaded and the Robust Multi-array Average (RMA) algorithm was used for background adjustment without quantile normalization (Irizarry et al., 2003). For array- or sequence-based data measured using the Illumina platform, processed data were directly downloaded. For sequence-based data from TCGA, the FPKM (fragments per kilobase of transcript per million fragments mapped) (Trapnell et al., 2010) value was downloaded.

For array-based data, the probe ID was mapped to the Entrez gene ID with the corresponding platform file. If a probe was mapped to zero or multiple genes, then data of this probe were discarded. If multiple probes were mapped to the same gene, the expression value of this gene was defined as the arithmetic mean of the values of those probes. For sequence-based data from ArrayExpress, gene symbols were mapped to Entrez gene ID with the biological database network (Mudunuri et al., 2009) (bioDBnet^3^). For sequence-based data from TCGA, Ensembl gene IDs were mapped to unique Entrez gene IDs of protein coding genes.

### Feature Selection

Based on gene expression profiles for CRC and normal colorectal tissues in training data (shown in Table 1), we selected gene pairs with reversal REOs by comparing highly stable gene pairs of CRC with normal tissue samples (the threshold was 90% in this study).

In the training datasets, the gene measurement of each gene was converted to the corresponding rank within each sample (i.e., the smallest measurement was converted to the minimum rank and the largest measurement was converted to the maximum rank). Then, pair wise comparisons were performed for all within-sample genes to identify stable gene pairs for a specific tissue type. For one sample, the REO pattern of two genes, *i* and *j*, was denoted as *G_*i*_* > *G*_*j*_ (or *G_*i*_* < *G*_*j*_) if the rank of gene *i* was higher (or lower) than that of gene *j*. If gene pair (*i*, *j*) had the same REO pattern as did a majority of samples (e.g., 90%), it was considered a stable REO pattern and the gene pair was defined as a stable gene pair. For two groups of samples, a gene pair with a stable but reversal REO pattern in the two groups was defined as a reversal gene pair. For the reversal gene pairs identified in the above process, REOs of gene pairs in CRC tissues were defined as the CRC signature that was applied for defining the incidence risk score of precancerous colorectal lesions.

### Risk Scoring Model Construction

In this study, we defined the REO patters *G_*i*_* > *G*_*j*_ and *G_*i*_* < *G*_*j*_ to characterize CRC and normal tissues, respectively. For each non-cancer patient with a precancerous lesion, the risk score was simply calculated as the percentage of REOs characterizing CRC. The incidence risk score for a particular sample was calculated as:

Score = *n*/*m*,

where *m* was the number of gene pairs included in the signature, *n* was the number of gene pairs with the same REO patterns characterizing CRC. The higher the incidence risk score was, the greater the cancer incidence risk that the patient had.

The performance of the risk score was then evaluated using samples from patients with precancerous colorectal lesions (including UC and adenomas) at different disease stages from multiple datasets (Table 2).

### Functional Analyses

A total of 244 pathways covering 6,934 unique genes were obtained from Kyoto Encyclopedia of Genes and Genomes (KEGG) database^4^ (Kanehisa and Goto, 2000). The hypergeometric distribution model was used to calculate the significance of enriched pathways with interested genes (Fury et al., 2006). And *p* values were adjusted with Benjamini-Hochberg method.

## Results

### Acquisition of REO Features

**FIGURE 1:**
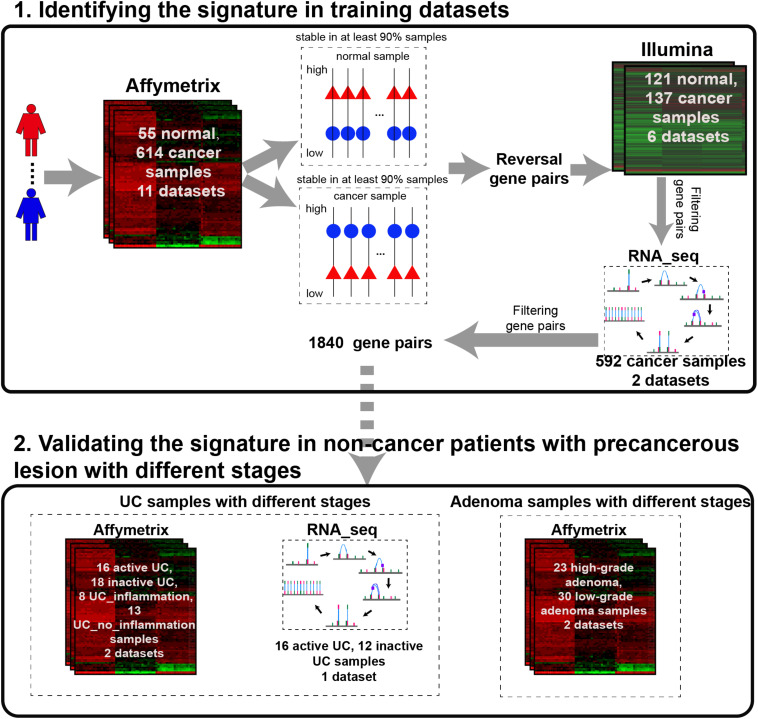
The analysis procedure for identifying CRC incidence-risk score.

The analysis procedure of this study is described in Figure 1. Considering that carcinogenesis of CRC is a continuous, multistep malignant transformation of normal colorectal tissues, we initially identified gene pairs with stable but reversal REOs between CRC and normal colorectal tissue samples with a threshold of 90% (see section “Material and Methods”).

For the 614 CRC and 55 normal colorectal tissue samples from the 11 datasets measured using the Affymetrix microarray platform (see Table 1), 356,573 gene pairs with stable (threshold of 90%) but reversal REO patterns between CRC and normal tissues were identified; these gene pairs were defined as reversal gene pairs. Similarly, for the 137 CRC and 121 normal colorectal tissue samples from the six datasets measured using the Illumina microarray platform (see Table 1), 406,957 reversal gene pairs were identified. We found 18,135 gene pairs that were consistently detected in the above two lists of reversal gene pairs. Among those 18,135 gene pairs, we further selected 1,840 gene pairs that had identical REO patterns in at least 90% of the 556 cancer samples in TCGA and 36 cancer samples in the GSE50760 dataset which were measured using the RNA_seq platform. Finally, the 1,840 gene pairs (see Supplementary Table S1) were selected and the REOs of the selected gene pairs of CRC tissues were defined as the CRC signature that was applied for defining the incidence risk score of precancerous colorectal lesions (see section “Material and Methods”).

### Validation and Functional Analysis of the CRC Incidence Risk Signature

For each sample, the risk score was simply calculated as the percentage of REOs characterizing CRC, which was close to 0 and 1 in normal and CRC tissues, respectively. Among training data with all merged samples, score medians were 0.0174 and 0.9891 for normal and CRC tissues, respectively. In the GSE22619 dataset of 10 normal colorectal tissues, we found the median score to be 0.1139. With RNA-seq, we studied 13 CRC tissue samples obtained via surgical resection and 33 CRC tissues obtained from biopsy (Guo et al., 2018), finding score medians of 0.9919 and 0.9271, respectively. This data indicated that the risk score was applicable for CRC tissues obtained for biopsy, although there were variations in data.

To elucidate the functions and pathways that were associated with the CRC incidence risk signature, KEGG pathway analysis were performed. Functional enrichment analysis of 1,580 genes included in the signature showed that 10 pathways were significantly enriched (*p* < 0.05, Supplementary Figure S1), including p53 signaling pathway and ECM-receptor interaction pathway. The p53 signaling pathway has been reported to be involved in cell cycle regulation and suppression of tumor expression (Hu et al., 2007; Kruiswijk et al., 2015; Tanikawa et al., 2017). ECM-receptor interaction pathway plays an important role in the process of CRC (such as tumor shedding, adhesion, degradation, movement and hyperplasia) and could promote the development of epithelial mesenchymal transition (EMT) in cancer cells (Rahbari et al., 2016). Moreover, ECM also plays a key role in the process of other cancer types, such as prostate cancer and gastric cancer (Andersen et al., 2018; Yan et al., 2018).

### The Performance of the CRC Incidence Risk Score in UC Samples

The typical pathogenesis of CRC is the transformation from normal cells to quiescent chronic inflammation. Dysplasia eventually arises from persistent inflammation and ultimately progresses to outright malignant transformation (Bjerrum et al., 2014). Thus, we evaluated the performance of our score in UC samples at different stages of the disease course.

**FIGURE 2:**
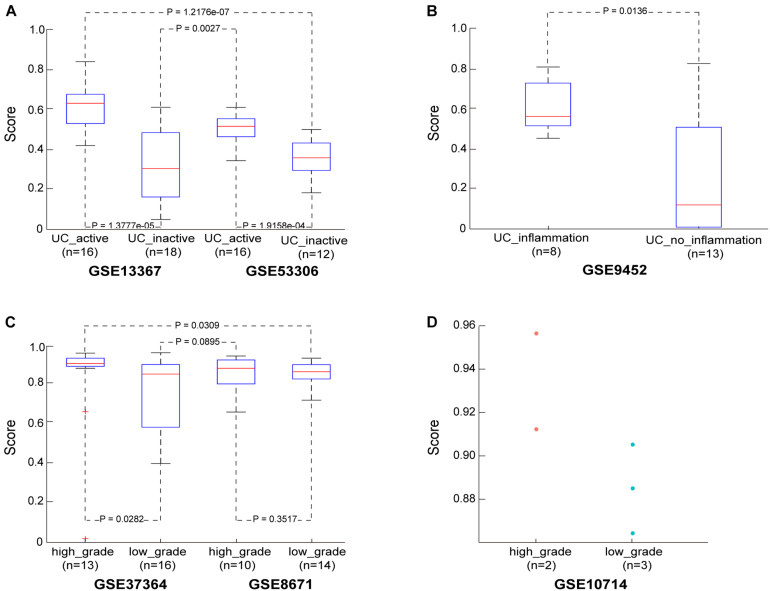
The performance of the CRC risk signature in UC and adenoma samples. **(A)** The score in the UC samples from dataset GSE13367 and GSE53306. **(B)** The score in the UC samples from dataset GSE9452. **(C)** The score in the adenoma samples from dataset GSE37364 and GSE8671. **(D)** The score in the adenoma samples from dataset GSE10714.

Then, in the GSE13367 dataset consisting of 16 active and 18 inactive (remissive) UC samples, the median CRC incidence risk score of active UC samples was 0.6402, significantly higher than that in remissive UC samples (Wilcoxon rank sum test; *p* = 1.3777e-05). Similar findings were also obtained in the GSE53306 dataset consisting of 16 active and 12 remissive UC samples (Wilcoxon rank sum test; *p* = 1.9158e-04). Detailed results are shown in Figure 2A and Supplementary Table S2. Importantly, the incidence risk scores of the 16 active UC samples from the GSE13367 dataset were also significantly higher than those in the 12 remissive UC samples from the GSE53306 dataset (Wilcoxon rank sum test; *p* = 1.2176e-07). Similar results were also obtained when analyzing active and remissive UC samples from datasets GSE53306 and GSE13367 (Wilcoxon rank sum test; *p* = 0.0027), respectively.

We also evaluated the applicability of our score to the GSE9452 dataset, which included 8 UC samples with and 13 UC samples without (UC_inflammation; UC_without_inflammation) macroscopic signs of inflammation. We found that CRC incidence risk scores in UC_inflammation samples (median = 0.5682) were significantly higher than those in UC_without_inflammation samples (median = 0.1228) (Wilcoxon rank sum test; *p* = 0.0136), as shown in Figure 2B and Supplementary Table S3. These findings further confirmed that our score could predict UC sample cancer incidence risk.

### The Performance of the CRC Incidence Risk Score in Adenoma Samples

The transformation of normal colorectal tissue to adenomatous tissue and finally to outright malignancy is the typical pathogenic process of CRC (Conteduca et al., 2013; Brenner et al., 2014). We thus also evaluated our score in colorectal adenoma samples at different disease stages.

In the GSE37364 dataset consisting of 13 high-grade and 16 low-grade dysplasia colorectal adenoma samples, the median incidence risk score in the high-grade dysplasia samples was 0.9076, significantly higher than that in the low-grade dysplasia samples (median = 0.8543) (Wilcoxon rank sum test; *p* = 0.0282). In the GSE8671 dataset, the CRC incidence risk scores in the 10 high-grade dysplasia samples (median = 0.8837) were only slightly higher than those in the 14 low-grade dysplasia samples (median = 0.8663, Wilcoxon rank sum test, *p* = 0.3517), as shown in Figure 2C and Supplementary Table S4, which may be ascribed to insufficient sample size. Then, we merged samples from the GSE37364 and GSE8671 datasets to further evaluate the performance of our score. For merged data with 23 high-grade and 30 low-grade dysplasia samples, the median CRC incidence risk score of high-grade dysplasia samples was 0.9027, significantly higher than that of low-grade dysplasia samples (median = 0.8565) (Wilcoxon rank sum test; *p* = 0.0191). The CRC incidence risk scores of the 13 high-grade dysplasia samples from the GSE37364 dataset were also significantly higher than those of the 14 low-grade dysplasia samples from the GSE8671 dataset (Wilcoxon rank sum test; *p* = 0.0309). Similar results were also obtained for high-grade dysplasia samples from the GSE8671 dataset and low-grade dysplasia samples from the GSE37364 dataset (Wilcoxon rank sum test; *p* = 0.0895). We also evaluated our score using the GSE10714 dataset consisting of 2 high-grade and 3 low-grade dysplasia colorectal adenoma samples. The median incidence risk score in the high-grade dysplasia samples was 0.9345, also higher than that in the low-grade dysplasia samples (median = 0.8853), as shown in Figure 2D.

Our aforementioned results further demonstrated that our score could well predict the incidence risk of CRC in non-cancer patients with precancerous lesions and that it was applicable to samples from different sources.

## Discussion

CRC mainly develops from malignant transformation of acquired precancerous lesions, such as IBD and colorectal adenomas. Based on the qualitative transcriptional characteristics, we developed a signature to assess the incidence risk of precancerous colorectal lesions to CRC by calculating the percentage of gene pairs in our signature that characterized the CRC tissue. For non-cancer patients with precancerous colorectal lesions, such as UC and adenomas, at different stages in the disease course and using data from multiple datasets, our score was well verified. Moreover, we also found that the CRC incidence risk scores of pan-colitis samples were higher than those of left-sided colitis samples, as was previously reported (Fujita et al., 2018).

The top five highest-frequency genes in the CRC incidence risk signature were *ABCG2, SLC51B, CLDN1, TEX11*, and *SLC25A34*, which have been reported to be related with the pathogenesis of CRC. *ABCG2* plays an important role in the progression and metastasis of CRC (Liu et al., 2010). The increased expression of *SLC51B* (also called *OST*β) in feces, one of the key membrane transporters of bile acids, is positively correlated with the incidence of CRC (Ballatori et al., 2009). Studies have suggested that expression of *CLDN1* is a prognostic factor in CRC patients (Nakagawa et al., 2011), while *TEX11* likely serves as a biomarker of early onset CRC (Luo et al., 2013). All of these genes are potential targets for future therapeutic interventions.

Due to the lack of corresponding clinical follow-up data, we could not verify whether individuals without cancer and with high CRC incidence risk score, as identified by our signature eventually develop cancer. In future research, we plan to collaborate with a tertiary healthcare facility to better evaluate UC or adenoma, to perform sampling at more diverse sites pre-malignant sites and samples at different stages of illness progression. Patients should be closely followed in the future to further evaluate the utility of our score and to compare the CRC incidence-risk score with the time from diagnosis to cancer incidence.

In summary, our score, based on qualitative transcriptional parameters, is robust against batch effects as well as amplification bias for minimum specimens. Thus, our calculated score is applicable to be used in the individualized diagnosis and is generally suitable for analyzing inaccurately sampled tissues in the clinical setting.

## Data Availability Statement

The datasets presented in this study can be found in online repositories. The names of the repository/repositories and accession number(s) can be found in the article/Supplementary Material.

## Author Contributions

QG, QZ, and WJ conceived the study, analyzed the data, made figures, performed the statistical analysis, and drafted the manuscript. JX, JC, HY, JH, and LA searched the data and participated in the statistical analysis. YX participated in discussing and revising the manuscript. ZG and GG conceived of the study, participated in its design and coordination, helped to draft the manuscript, and supervised the work. QG, QZ, and LA revised the manuscript. All authors read and approved the final manuscript.

## Conflict of Interest

The authors declare that the research was conducted in the absence of any commercial or financial relationships that could be construed as a potential conflict of interest.

## Footnotes

1 http://www.ncbi.nlm.nih.gov/geo/

2 http://cancergenome.nih.gov/

3 https://biodbnet-abcc.ncifcrf.gov/db/db2db.php

4 https://www.kegg.jp/kegg/pathway.html

## Supplementary Material

Supplementary Figure 1 Bar plot of KEGG pathways enriched by genes involved in the CRC risk signature.

Supplementary Table 1 The gene pair of the CRC incidence-risk score signature.

Supplementary Table 2 CRC incidence risk scores in active and inactive (remissive) UC samples.

Supplementary Table 3 CRC incidence risk scores in UC_inflammation and UC_without_inflammation samples.

Supplementary Table 4 CRC incidence risk scores in high-grade and low-grade dysplasia adenoma samples.

## Abbreviations

CRC: Colorectal Cancer
UC: Ulcerative Colitis
REO: Relative Expression Ordering
IBD: Inflammatory Bowel Disease
CD: Crohn’s Disease
RMA: the Robust Multi-array Average
FPKM: Fragments Per Kilobase of transcript per Million fragments.
