# Supplementary figures and images for: A Qualitative Transcriptional Signature for the Risk Assessment of Precancerous Colorectal Lesions
Source: Front Genet. 2021 Jan 15;11:573787. doi: 10.3389/fgene.2020.573787 (PMC7844367; doi:10.3389/fgene.2020.573787)

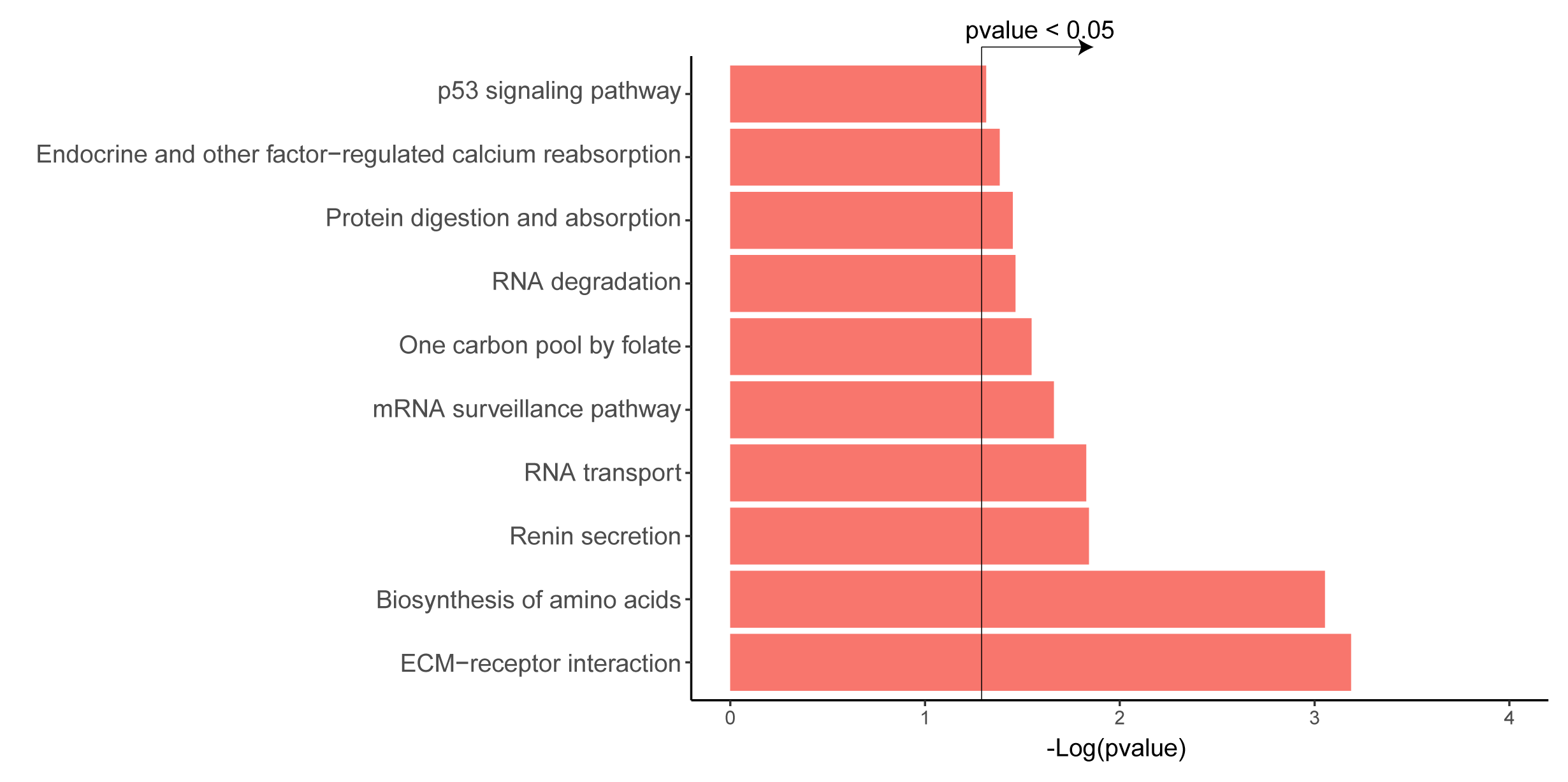

Supplement: Supplementary Figure 1 — Bar plot of KEGG pathways enriched by genes involved in the CRC risk signature. [file Image_1.TIF]
